# Coding-Complete Genome Sequence and Phylogenetic Relatedness of a SARS-CoV-2 Strain Detected in March 2020 in Cameroon

**DOI:** 10.1128/MRA.00093-21

**Published:** 2021-03-11

**Authors:** Richard Njouom, Serge Alain Sadeuh-Mba, Jules Tchatchueng, Moussa Moïse Diagne, Ndongo Dia, Paul Alain Ngoupo Tagnouokam, Yap Boum, Achta Hamadou, Linda Esso, Ousmane Faye, Mathurin Cyrille Tejiokem, Marie Claire Okomo, Alain Etoundi, Elisabeth Carniel, Sara Eyangoh

**Affiliations:** a Centre Pasteur du Cameroun, Yaounde, Cameroon; b Institut Pasteur de Dakar, Dakar, Senegal; c Epicentre, Yaounde, Cameroon; d Public Health Emergency Operation Center, Ministry of Health, Yaounde, Cameroon; e National Public Health Laboratory, Yaounde, Cameroon; DOE Joint Genome Institute

## Abstract

We describe the coding-complete genome sequence of a severe acute respiratory syndrome coronavirus 2 (SARS-CoV-2) strain obtained in Cameroon from a 58-year-old French patient who arrived from France on 24 February 2020. Phylogenetic analysis showed that this virus, named hCoV-19/Cameroon/1958-CMR-YAO/2020, belongs to lineage B.1.5 and is closely related to an isolate from France.

## ANNOUNCEMENT

A novel coronavirus, recently named severe acute respiratory syndrome coronavirus 2 (SARS-CoV-2), that is related to the genus *Betacoronavirus* and the family *Coronaviridae* was identified in the city of Wuhan, China, in December 2019 (1). As of 30 December 2020, Cameroon has recorded 26,848 confirmed cases, 448 deaths (case fatality rate, 1.7%), and 25,468 recoveries (95%) (2). As of 30 December 2020, 294,185 complete genome sequences of SARS-CoV-2 have been obtained and deposited in the GISAID database (3). Phylogenetic analysis showed that these viruses diversified during the duration of the pandemic into two major lineages, A and B, with several sublineage diversifications (4). A recent study indicated that the African SARS-CoV-2 sequences also diversified into two lineages, A and B, with B being more diverse with multiple sublineages (5). Here, we report the coding-complete genome sequence of a SARS-CoV-2 strain obtained from a sample taken on 5 March 2020 in Cameroon from a 58-year-old French patient who arrived from France on 24 February 2020. Ethical clearance to conduct this study was obtained from the Cameroon National Ethics Committee (approval number 2020/05/1224/CE/CNERSH/SP).

Diagnosis of coronavirus disease 2019 (COVID-19) was conducted at the Centre Pasteur du Cameroun (Yaounde, Cameroon) by real-time reverse transcription (RT)-PCR (6). The nasopharyngeal swab sample obtained from the patient had cycle threshold (*C_T_*) values of 31.9 for the E gene and 33.4 for the RdRP gene and was further sent to the Institut Pasteur de Dakar (Dakar, Senegal) for the first confirmatory diagnosis and sequencing. Briefly, viral RNA was extracted using the QIAamp viral RNA minikit and amplified by RT-PCR as described previously (7). The PCR products were purified and the DNA concentrations were measured with a Qubit 3 fluorometer (Invitrogen). DNA products (multiplex PCR pools A and B) were pooled in equal concentrations. DNA libraries were generated from the pooled amplicons using the Illumina DNA preparation kit according to the manufacturer’s specifications. Whole-genome sequencing was performed with paired-end reads using the Illumina MiSeq reagent kit v3 (150 cycles) on an Illumina MiSeq instrument. The 187,596 reads were trimmed for quality and length and assembled by mapping to the reference genome from Wuhan (GenBank accession number NC_045512.2) using the already published USAMRIID-WRAIR pipeline (8). All tools were run with default parameters unless otherwise specified. Multiple sequence alignment and pairwise sequence comparisons with the hCoV-19/Cameroon/1958-CMR-YAO/2020 sequence obtained were carried out with CLC Main Workbench v5.7.2 software. Estimation of the best-fitting substitution model (GTR+I+Γ4) was conducted with Smart Model Selection (9) based on the Bayesian information criterion. Inference of the phylogenetic tree was conducted with the maximum likelihood approach using PhyML v3.0 (10) with subtree pruning and regrafting (SPR) branch swapping. The reliability of the tree topology was estimated with 1,000 bootstrap replicates.

**FIG 1:**
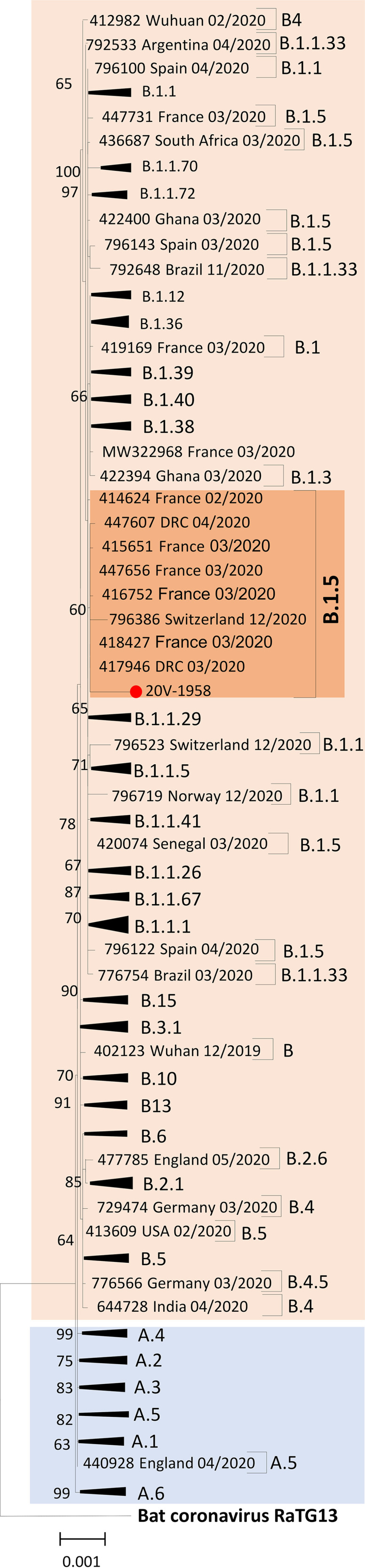
Phylogenetic analysis of the Cameroon SARS-CoV-2 complete genome sequence generated in this study (indicated with a red circle), with complete sequences from different countries available in the GISAID database as of 30 December 2020. Multiple sequence alignment and pairwise sequence comparisons were carried out with CLC Main Workbench v5.7.2 software. Inference of the phylogenetic tree was conducted by the maximum likelihood approach using PhyML v3.0 with SPR branch swapping. The reliability of the tree topology was estimated with 1,000 bootstrap replicates.

The consensus sequence length was 29,686 bp, corresponding to 99.25% of the genome, with a GC content of 33.02%. The amino acid substitutions found in the hCoV-19/Cameroon/1958-CMR-YAO/2020 virus, compared with the reference, were M protein L206V, M protein N207D, nonstructural protein 6 (NSP6) L227F, and NSP12 P323L. The maximum likelihood phylogenetic tree (Fig. 1) shows that hCoV-19/Cameroon/1958-CMR-YAO/2020 clusters with B.1.1.5 SARS-CoV-2 lineage sequences isolated in France in March 2020.

The report of a SARS-CoV-2 coding-complete genome from Cameroon will allow investigators to follow the spread of this strain in the country and to evaluate its potential accumulation of mutations over time, improving our understanding of the molecular epidemiology of SARS-CoV-2 in Cameroon.

### Data availability.

The viral sequence from the patient was deposited in the GISAID EpiCoV SARS-CoV-2 database with the following identifiers: virus name, hCoV-19/Cameroon/1958-CMR-YAO/2020; GISAID accession number, EPI_ISL_512873; NCBI GenBank accession number, MW566800. The raw reads were submitted to the NCBI Sequence Read Archive (SRA) under BioProject accession number PRJNA701254 and SRA accession number SRR13677539.
